# Anthrax Toxins Induce Shock in Rats by Depressed Cardiac Ventricular Function

**DOI:** 10.1371/journal.pone.0000466

**Published:** 2007-05-23

**Authors:** Linley E. Watson, Shu-ru Kuo, Khurshed Katki, Tongyun Dang, Seong Kyu Park, David E. Dostal, Wei-Jen Tang, Stephen H. Leppla, Arthur E. Frankel

**Affiliations:** 1 Division of Cardiology, Scott and White Memorial Hospital, Scott, Sherwood and Brindley Foundation, Temple, Texas, United States of America; 2 Department of Medicine, Texas A&M University System, Health Science Center College of Medicine, Temple, Texas, United States of America; 3 Scott and White Cancer Research Institute, Temple, Texas, United States of America; 4 Division of Molecular Cardiology, Texas A&M University System, Health Science Center College of Medicine, and Central Texas Veterans Health Care System, Temple, Texas, United States of America; 5 Ben May Institute for Cancer Research, The University of Chicago, Chicago, Illinois, United States of America; 6 Bacterial Toxins and Therapeutics Section, National Institutes of Allergy and Infectious Diseases, Bethesda, Maryland, United States of America; Columbia University, United States of America

## Abstract

Anthrax infections are frequently associated with severe and often irreversible hypotensive shock. The isolated toxic proteins of *Bacillus anthracis* produce a non-cytokine-mediated hypotension in rats by unknown mechanisms. These observations suggest the anthrax toxins have direct cardiovascular effects. Here, we characterize these effects. As a first step, we administered systemically anthrax lethal toxin (LeTx) and edema toxin (EdTx) to cohorts of three to twelve rats at different doses and determined the time of onset, degree of hypotension and mortality. We measured serum concentrations of the protective antigen (PA) toxin component at various time points after infusion. Peak serum levels of PA were in the µg/mL range with half-lives of 10–20 minutes. With doses that produced hypotension with delayed lethality, we then gave bolus intravenous infusions of toxins to groups of four to six instrumented rats and continuously monitored blood pressure by telemetry. Finally, the same doses used in the telemetry experiments were given to additional groups of four rats, and echocardiography was performed pretreatment and one, two, three and twenty-four hours post-treatment. LeTx and EdTx each produced hypotension. We observed a doubling of the velocity of propagation and 20% increases in left ventricular diastolic and systolic areas in LeTx-treated rats, but not in EdTx-treated rats. EdTx-but not LeTx-treated rats showed a significant increase in heart rate. These results indicate that LeTx reduced left ventricular systolic function and EdTx reduced preload. Uptake of toxins occurs readily into tissues with biological effects occurring within minutes to hours of serum toxin concentrations in the µg/mL range. LeTx and EdTx yield an irreversible shock with subsequent death. These findings should provide a basis for the rational design of drug interventions to reduce the dismal prognosis of systemic anthrax infections.

## Introduction

*Bacillus anthracis* is a spore-forming, Gram-positive bacterium and is the causative agent of anthrax. Infection by inhalation of *B. anthracis spores* can result in a mortality rate of up to 80% [1]. Despite a long history *of Bacillus anthracis* infections as a cause of human and animal disease and its notoriety as an agent of biological warfare, exactly how the bacteria kill the host is unclear. Although aggressive antibiotic therapy can prevent bacterial growth, infected individuals still die, most probably due to high concentrations of anthrax toxins already accumulated in the body [2].

*Bacillus anthracis* vegetative bacteria secrete three proteins—protective antigen (PA), lethal factor (LF), and edema factor (EF) which combine to form anthrax lethal toxin (LeTx; PA and LF) and anthrax edema toxin (EdTx; PA and EF) [3]. PA binds receptors tumor endothelial marker 8 (TEM8) and capillary morgenesis gene-2 (CMG2) on normal tissues, undergoes cell surface furin-mediated endoproteolytic cleavage and release of a twenty kilodalton fragment, oligomerization of the remaining sixty-three kilodalton PA portions to form heptamers, followed by binding of three molecules of LF or EF per heptamer [4]. The toxin-receptor complex is depalmitoylated, associates with co-receptors including low-density lipoprotein receptor-related protein 6 (LRP6), migrates into lipid rafts, is E3 ubiquitin ligase Cbl-ubiquinated, associates with Eps15 and then undergoes clathrin-mediated endocytosis [5]. After acidification in endosomes, the PA 2β2-2β3 strands unfold, insert into the endosomal membranes, dissociate from the CMG2 and TEM8 receptors, and create a 14-member β-barrel pore [6]. EF and LF bind to the pore entrance and translocate to the cytosol with a cytosolic translocation factor chaperone [7]. In the cytosol, LF is a Zn^2+^-metalloprotease which specifically cleaves the NH_2_-termini of mitogen-activated protein kinase kinases (MEKs) resulting in their inactivation [8]. Intoxicated cells lose normal mitogen-activated kinase pathway signaling. EF is a calmodulin-dependent adenylyl cyclase that elevates intracellular levels of cAMP and produces altered cell physiology [9]. Evidence for the central role of the toxins in anthrax pathogenesis includes the lethality of purified preparations of LeTx and EdTx in rodents [10], [11], the thousand-fold reduction in toxicity of bacterial strains with mutated, inactivated anthrax toxin genes [12] and the protection of animals from death due to *Bacillus anthracis* infection by prophylactic treatment with antibodies or vaccines to anthrax toxins [13], [14].

In the 2001 bioterrorism anthrax experience, patients with inhalational anthrax developed refratory hypotension [15], [16]. In part due to the acuteness and severity of the events, full hemodynamic and molecular studies were not conducted. In contrast to lipopolysaccharide-induced endotoxic shock, excessive inflammatory cytokine and nitric oxide release do not appear to contribute to the circulatory shock and lethality occurring with anthrax toxins in rodent models [10], [11], [17], [18]. These results imply a possible complex pathophysiology for anthrax associated shock. The purpose of this study was to determine the acute cardiac and vascular hemodynamic effects of LeTx and EdTx in the rat and demonstrate that many of abnormalities are consistent with human inhalation anthrax pathophysiology.

Newer methods used in this study to dissect the pathophysiology of shock in unrestrained rodents include instrumentation with intra-aortic catheter probes connected to telemetry units and transthoracic echocardiography with state-of-the-art ultrasound technology. These methods permit dissection of the contribution of the heart, blood volume and blood vessels to cardiac output and blood pressure.

## Results

### Effects of LeTx and EdTx Dosage on Mortality

**Figure 1:**
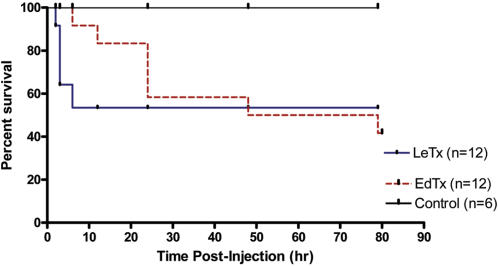
Survival of Sprague Dawley rats (250 to 300 g) treated with single intravenous bolus infusion of LeTx (0.03 mg PA+0.015 mg LF) or EdTx (0.15 mg PA+0.075 mg EF) and monitored for two weeks. These are the same doses used for telemetry and ECHO experiments. N = 12 for each experiment. Kaplan-Meier graphs prepared.

Examination of toxicity in Sprague Dawley rats was performed by an intravenous injection of different doses of LeTx and EdTx and mortality (Table 1). Survival curves for rats treated with single intravenous bolus infusion of LeTx (0.03 mg PA+0.015 mg LF) or EdTx (0.15 mg PA+0.075 mg EF) is shown in Figure 1. All of the LeTx deaths occurred within the first 6 hr, whereas the last EdTx death occurred after 72 hr. The Kaplan-Meier survival of LeTx-treated rats, compared to control is not significantly different (p = 0.08 and Fisher's exact p = 0.11). Kaplan-Meier survival of controls versus EdTx is statistically significant (p. value = 0.047 and Fisher's exact p = 0.038). We chose doses for further studies of LeTx (0.03 mg PA+0.15 mg LF) and EdTx (0.15 mg PA and 0.075 mg EF) which yielded 58% survival and 42% survival, respectively.

**Table 1:** Mortality of Anthrax Toxins*

Toxin	Dose (PA/LF or EF)	Mortality (%)
LeTx	0.04 mg/0.02 mg	100 (n = 3)
	0.032 mg/0.016 mg	66 (n = 3)
	0.03 mg/0.015 mg	42 (n = 12)
	0.024 mg/0.012 mg	33 (n = 3)
EdTx	0.15 mg/0.075 mg	58 (n = 12)
	0.075 mg/0.0375 mg	0 (n = 3)
	0.04 mg/0.02 mg	0 (n = 3)

* n = the number of animals used in each dose study

### Serum Levels of Anthrax toxins

**Figure 2:**
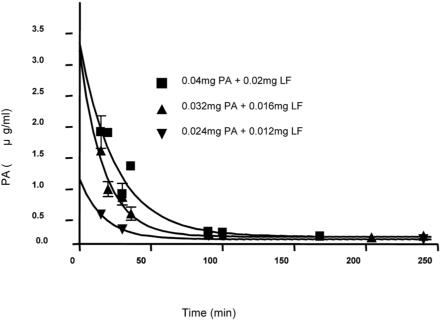
Serum levels of PA after single bolus intravenous infusions of different doses of LeTx to Sprague Dawley rats (250 to 300 g). Concentrations of toxin components measured by ELISA as described in the text. Peak concentrations and half-lives shown in Table 2.

To determine half-life and plasma levels of PA following injection, rats were administered various doses of LeTx (2:1 ratios of PA/LF) and EdTx (2:1 ratios of PA/EF) via the tail-vein injection. The LeTx was given as single bolus infusions using doses of 0.024 mg PA+0.012 mg LF, 0.03 mg PA+0.015 mg LF, 0.032 mg PA+0.016 mg LF, 0.04 mg PA+0.02 mg LF in 500 µL phosphate-buffered saline (PBS). EdTx was given as single bolus infusions of 0.15 mg PA+0.075 mg EF in 500 µL PBS. After various times (0–250 min) blood samples were collected and plasma levels of PA were analyzed using ELISA, as described in the methods. As shown in Table 2 and Figure 2, higher doses of anthrax toxin resulted in increased serum concentrations of PA. Peak levels were in the µg/mL range and half-lives were 10–20 minutes.

**Table 2:** Anthrax toxins in serum samples*

Toxin	Dose (PA/LF or EF)	Half-life of PA (min)	Co (µg/ml) of PA
LeTx	0.04 mg/0.02 mg	17	2.9
	0.032 mg/0.016 mg	12	2.8
	0.03 mg/0.015 mg	12	1.4
	0.024 mg/0.012 mg	12	0.9
EdTx	0.15 mg/0.075 mg	23	11.4

* Three animals were used in each dose study; ND, not determined.

### Blood Pressure Telemetry Measurements

**Figure 3:**
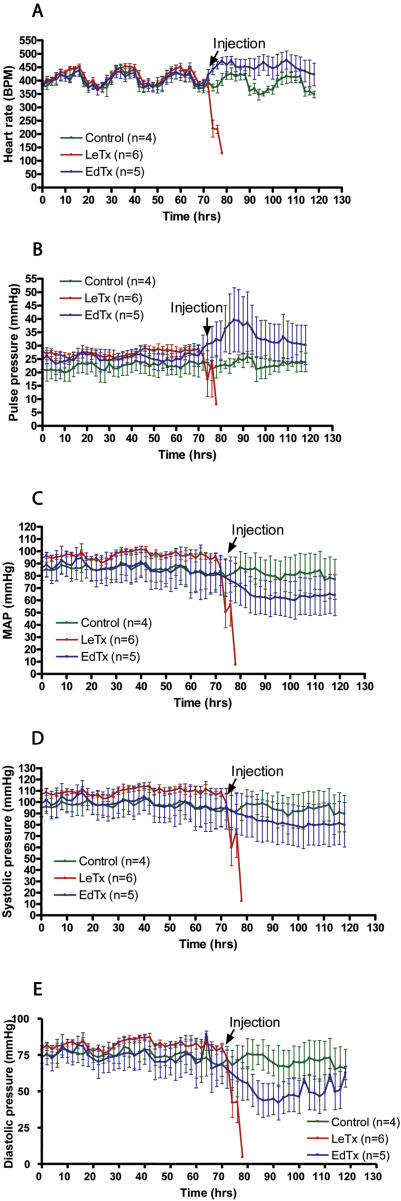
Hemodynamic parameters measured by telemetry of conscious Sprague Dawley rats (250g to 300g) treated with single intravenous bolus infusions of LeTx (0.03 mg PA+0.015 mg LF) or EdTx (0.15 mg PA+0.075 mg EF). A. Heart rate (beats per min), B. Pulse pressure (mmHg), C. Mean arterial pressure (mmHg), D. Systolic blood pressure (mmHg), and E. Diastolic blood pressure (mmHg) were measured every 15 min. for 72 hr before and after toxin administration.

Hemodynamic parameters were measured by telemetry of conscious Sprague Dawley rats that were treated with single intravenous bolus infusions of toxins. Radiofrequency measurements of baseline heart rate and blood pressures were recorded for 72 hr, after which rats were administered tail-vein injections of LeTx (0.03 mg PA+0.015 mg LF), EdTx (0.15 mg PA+0.075 mg EF) or PBS (control). Bradycardia and hypotension developed in rats within 1 hr after exposure to LeTx (Figure 3). The rapid onset of bradycardia and decrease in systolic and diastolic pressure is consistent with a primary effect of LeTx on the heart. Although rats administered EdTx demonstrated decreases in systolic, diastolic and mean arterial pressures after 18–48 hr, an increase in heart rate occurred (Figure 3). The increase in heart rate may represent compensation for the lowered blood pressure. There was a widened pulse pressure and reduction in diastolic blood pressure consistent with reduced systemic vascular resistance and reduced vascular tone.

### Effects of LeTx and EdTx on Cardiac Function

**Figure 4:**
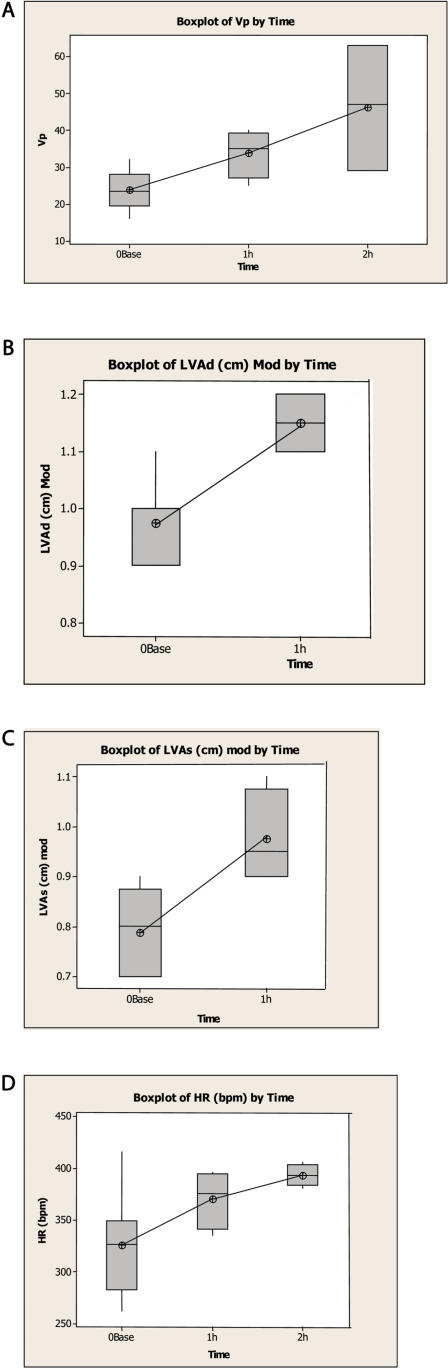
Boxplots at different times post anthrax toxin bolus iv infusion of echocardiographic parameters: A. LeTx treated rats at zero, one and two hr post-infusion, velocity of propagation was 24±5, 34±7 and 46±17 cm/sec, respectively, with significant difference (P = 0.05 at one hr compared to controls and P = 0.007 at two hr compared to controls); B. LeTx treated rats at zero and one hr post-infusion, left ventricular diastolic area was 0.98±0.07 and 1.15±0.06, respectively, with significant difference (P = 0.01); C. LeTx treated rats at zero and one hr post-infusion, left ventricular systolic area was 0.79±0.08 and 0.98±0.10 cm, respectively, with significant difference (P = 0.02); D. EdTx treated rats at zero, one and two hr post-infusion, heart rate was 326±49, 371±28, and 393±10 beats per min, respectively, with significant difference (P = 0.05 at one hr and P = 0.001 at two hr compared to controls). The box represents the middle 50% of the data. The line through the box represents the median. The line (whiskers) extending from the box represent the upper and lower 25% of the data. The line of each plot connects the means of the sample.

Echocardiographic parameters including velocity of propagation (Vp), left ventricular diastolic area (LVAd), left ventricular systolic area (LVAs), heart rate were monitored at different times post anthrax toxin bolus iv infusion (Figure 4). LeTx treated rat hearts showed significant changes at one and two hrs with increased Vp, increased LVAd, and increased LVAs (Figure 4A, B, and C). Vp was 24±5, 34±7 and 46±17 cm/sec at 0, 1 and 2 hrs, respectively, with significant difference (P = 0.05 at one hr compared to controls and P = 0.007 at two hr compared to controls). LVAd was 0.98±0.07 and 1.15±0.06 at 0 and 1 hour, respectively, with significant difference (P = 0.01). LVAs was 0.79±0.08 and 0.98±0.10 cm at 0 and 1 hr, respectively, with significant difference (P = 0.02). These changes indicate LeTx induced myocardial effects. The Vp doubled by two hr and LVAd and LVAs increased by 20% in one hr. These results suggest that increased left ventricular end-systolic volume precedes the reduction in ejection fraction.

EdTx treated rat hearts showed significant increases in heart rate compared to controls at one and two hr post-injection (Figure 4D). Heart rate was 326±49, 371±28, and 393±10 beats per min at 0, 1 and 2 hours, respectively, with significant difference (P = 0.05 at one hr and P = 0.001 at two hr compared to controls). At one hour, rats treated with EdTx failed to show the changes in velocity of propagation or ventricular areas observed with LeTx (data not shown), consistent with a decrease in preload. In addition, there were no significant differences between controls and EdTx injected rats for ejection fraction, fractional shortening, and corrected velocity of circumferential fiber shortening suggesting EdTx has no direct myocardial effects. Taken together, the EdTx-induced sinus tachycardia is an expected early response to reduced preload.

## Discussion

The studies presented here provide the first detailed analyses of cardiovascular toxicity induced by purified anthrax toxins in a rat model. Both toxins induce significant hypotension. Importantly, LeTx and EdTx appear to produce the hypotension by affecting different parts of the cardiovascular system. Serum levels of toxins were measurable in all tested rats. Lethality and hypotension were associated with doses of toxins that produced peak PA levels in the µg/mL range. This is consistent with the measurements of toxin levels in spore-infected animals [2], [19]. Hypotension and death occurred within hr of reaching similar ranges of toxin in the blood of infected rats, rabbits and guinea pigs.

LeTx rapidly induced acute myocardial dysfunction similar to that observed with fulminant myocarditis with significant increases in the LV systolic area, LV diastolic area, and ventricular compliance. Left ventricular dilatation was expected from acute myocardial dysfunction such as fulminant myocarditis. Increased ventricular compliance is implied by the increased Vp. Ogawa reported that Vp was positively correlated with stroke volume, heart rate and left ventricular compliance [20]. Since stroke volume and heart rate did not significantly change, then increased ventricular compliance is the likely explanation for increases in Vp. Increased left ventricular compliance may be due to loss of functional intercalated discs that connect the myocardial cells. In contrast, EdTx has an intravascular volume effect. This observation is compatible with “third spacing” or decreased intravascular volume due to rapid fluid shifts out of the blood vessels.

Both observations are consistent with published results of toxin-induced rodent hypotension. Mice intravenously infused with LeTx developed malaise and death by 60–100 hr [10], [18], [21]. Corticosteroids and aldosterone were unable to prevent lethality or shock. Histopathology showed hypoxic tissue necrosis in liver, marrow, spleen and heart, pleural and peritoneal fluid and edema. There were elevations of serum erythropoietin and transaminase and hypoalbuminemia, hypofibrinogenemia, thrombocytopenia, elevated prothrombin time and partial thromboplastin time, and disseminated fibrin deposition and hemorrhage. Rats treated with LeTx died after two to twenty-one hr with refractory hypotension, bradycardia, lactic acidosis, and pleural effusions [13], [17], [22], [23]. Again, these results are consistent with shock and hypoxic secondary tissue injury. Mice treated with EdTx died with one to three days with hypotensive shock and “third spacing” in the intestinal lumen and other tissues [11]. Pathology showed fluid accumulation in intestines, adrenal hemorrhage, lymphocytolysis, osteoblast necrosis, and elevated transaminases and urea nitrogen. These results coincide with our observation of a reduction in preload due to a loss of vascular volume and tone. Additional studies in anthrax toxin-treated rats will be performed in our laboratory to better investigate the cardiac and vascular anatomy.

Previous reports support a critical role for mitogen activated protein kinase (MAPK) signaling in the heart. Tissue culture studies with antisense molecules and knockout mice show cardiomyocyte injury with impaired MEK1/2-ERK1/2 kinase activities [24]. Cancer patients treated with a small molecular weight MEK inhibitor develop heart failure [25]. Similarly, increases in vascular endothelial cAMP from calcitonin gene-related peptide G-protein activation led to decreased vascular resistance in hepatic, coronary, skin and gastric vessels [26]. Thus, the cAMP pathway is important in vascular homeostasis. Additional studies with tissues from anthrax toxin treated animals will be needed to confirm changes in these vital tissue-signaling pathways.

The observed cardiovascular defects induced by anthrax toxins were dependent on the presence of two toxin components (PA+LF or PA+EF). Rats received IV infusion of LF alone survived and showed no symptom of hypotension (data not shown). Previous studies have demonstrated that, in the absence of PA, injection of EF does not cause any symptom in treated animals [11], [27]. In addition, only combination of PA with LF or EF, but not LF or EF alone, displays toxicity in macrophage cells [28] and suppresses T lymphocyte activation [29].

Combinations of LeTx and EdTx as occurs in systemic anthrax infections would be predicted to generate more severe and irreversible hypotension due to an inability of either the heart or blood vessels to respond to lesions in the other organ. Preliminary studies with combinations of EdTx and LeTx show worse hypotension and greater lethality [23]. Examination of the hemodynamic parameters described in the current study with rats treated with mixtures of LeTx and EdTx will be useful to confirm these predictions.

In summary, these results suggest that the pathophysiology of anthrax shock may be closely linked with circulating toxins and involve both a rapid reduction in left ventricular preload and systolic function. Intervention to rescue animals and patients with anthrax shock will need to address both of these cardiovascular lesions. Rodents and other species provide an excellent system to test new therapeutics for this devastating disease.

## Materials and Methods

### Toxins

Anthrax toxin components PA, LF and EF were produced at over 95% purity with low endotoxin level as previously described [30]–[32], [28]. All toxin preparations were diluted in 1X phosphate-buffered saline (PBS), filter-sterilized, frozen and thawed only once prior to use. In each case, we used twice the amount of PA compared to LF or EF.

### Animals

Male Sprague-Dawley rats weighing between 180 and 230 g were purchased from Charles River Laboratories (Cambridge, MA) and allowed a one-week acclimation period after arrival to the animal facilities at Scott and White before experimentation or surgical procedures. Rat weights at the time of experiments were 250 to 300 g. Animals were housed two per polycarbonate tub with wood shavings and allowed food (Purina Rat Chow) and tap water *ad libitum*. The colony room lights were regulated on a 12:12-h light-dark cycle. Care and use of animals were designed in accordance with National Institutes of Health and American Association for the Accreditation of Laboratory Animal Care (AAALAC) guidelines, and approved by the Scott and White Memorial Hospital/Texas A and M University System Health Science Center Institutional Animal Care and Use Committee. Rats received bolus intravenous 500 µL infusions of sterile PBS, LeTx or EdTx.

### Survival Curves

Groups of three to twelve animals received intravenous injections of various doses of LeTx, EdTx, or toxin subcomponent. Animals were monitored post-injection hourly for eight hours and then every twelve hr for activity, posture, eating and drinking, and hair condition. Moribund animals were euthanized. Kaplan-Meier survival curves were compared by the log-rank test.

### Measurement of Anthrax Toxin Pharmacokinetics

Clearance of PA from the circulation of Sprague Dawley rats was determined following intravenous tail-vein injection of toxin into three rats. Tail-vein blood samples (100–200 µL) were collected at 15, 20, 30, 90, 100, 168, 240 and 1440 min-post injection, after which serum was harvested by sedimentation (14,000 rpm, 10 min, 22°C). Aliquots of serum were stored at−80°C until assayed. ELISA plates (Corning Costar 9018, Acton, MD) were incubated (16 hr, 4°C) with capture anti-PA antibody (2 µg/mL of 14B7) [33]. After wells were washed (PBS plus 0.05% Tween-20) and blocked (200 µL 5% non-fat milk) for one hour, 100 µL of serum sample (in 1∶20 and 1∶100 dilution) or standard (0 to 1 µg/mL of PA in 1:20 dilution of rat serum) in PBS containing 1% bovine serum albumin was added and incubated for one hr at room temperature. Plates were rewashed and reacted for two hr with 100 µL of 1 µg/mL biotinylated detection anti-PA antibody (8240, Abcam, Cambridge, MA). Biotinylation of anti-PA detection antibodies was prepared following the recommendations of the manufacturer (Pierce 21450 Kit, Pierce Biotechnology, Rockford, IL). After washing, 100 µl of horseradish peroxidase-conjugated streptavidin (1:8000, R&D Systems, Minneapolis, MN) was added and incubated for 1 hr. Wells were washed and incubated with 100 µL R&D substrate reagent 895000 for 20 min. Color development was stopped with 100 µL 2N H_2_SO_4_ and absorbance measured at 450 nm on a VERSAmax microplate reader (Molecular Devices, Sunnyvale, CA). Each test was performed in triplicate and the average of the data points was plotted using GraphPad Prism 4 (GraphPad, San Diego, CA). Curved-fits were calculated with one phase exponential decay equation: Y = Span (e^−kX^)+Plateau, k is elimination rate constant. The PA assay had a range of 3.91 ng/mL to 500 ng/mL with the inter-assay and intra-assay coefficients of variation less than 15%.

### Telemetry Measurements

Radio frequency devices were implanted for independent monitoring of blood pressure or heart rate using devices from Data Sciences Int. (St. Paul, MN) as described [34]. Briefly, rats randomized into control (N = 4), LeTx (N = 6) and EdTx (N = 5) groups were anesthetized with ketamine-xylazine, after which transmitters were implanted in the peritoneal cavity and attached pressure catheters were placed in the aortic lumen via the right carotid. Rats were allowed to recover from surgery for one week, after which baseline pressures were obtained for three days. Following tail-vein injection, recordings were made. The blood pressure recordings among groups were compared before and after exposure to LeTx and EdTx. Rats were administered 0.5 ml bolus of vehicle (PBS, control), LeTx (0.03 mg PA+0.015 mg LF) or EdTx (0.15 mg PA+0.075 mg EF) by tail-vein injection. Telemetric blood pressure and heart rate were recorded up to 72 hr.

### Echocardiography Measurements

Echocardiography was used to determine effects of LeTx and EdTx on cardiac function. At 12–24 hr prior to toxin administration, rats were subjected to echocardiography to establish baselines and exclude any animals with abnormal cardiac function. We used a previously established echocardiography protocol [35] to determine systolic and diastolic function in the rats. Briefly, prior to echocardiography, rats were anesthetized by administering an intramuscular injection of xylazine (10 mg/mL)-ketamine (100 mg/mL) cocktail in 0.1 ml. Heart rates were recorded at the beginning of echocardiography from the ECG monitor. Echocardiographic monitoring was performed using a General Electric Vivid I (Fairfield, CT). Transthoracic echocardiographic parameters were measured and calculations performed according to the American Society of Echocardiography guidelines [36]. All measurements were performed online using optimal digital images selected by the sonographer from more than 10 cardiac cycles. Left ventricular (LV) end-systolic and end-diastolic areas were traced in single-plane apical 4-chamber view and online Simpson's rule ejection fraction was calculated using the modified single-plane method. Standard formulae were used for echocardiographic calculations [37]. Rats randomized into control, LeTx, and EdTx treatment groups. For experiments, conscious rats were administered 0.5 ml bolus of vehicle (PBS, control), LeTx (0.03 mg PA+0.015 mg LF) or EdTx (0.15 mg PA+0.075 mg EF) by tail-vein injection. Each group had echocardiography performed at one, two, three and twenty-four hr post-injection.

### Statistical Analysis

Data were averaged and shown as means±standard error of mean (S.E.). Two sample differences were analyzed using Student's unpaired t test. All calculated P values were two tailed, and a value of P<0.05 was considered to indicate statistical significance. For rats that died overnight, statistical analysis was based on the last observed post-infusion time point.
